# BCR‐ABL enhances the prolyl isomerase activity of Pin 1 by interacting with DAPK1 in ph^+^ ALL

**DOI:** 10.1002/cam4.1478

**Published:** 2018-04-17

**Authors:** Wen‐bin Cao, Jian‐feng Yao, Si‐zhou Feng, Yi He, Er‐lie Jiang, Rong‐li Zhang, Dong‐lin Yang, Ming Gong, Xiao‐hui Zheng, Shu‐lian Chen, Jia‐li Sun, Lu‐kun Zhou, Ming‐zhe Han

**Affiliations:** ^1^ Hematopoietic Stem Cell Transplantation Center Institute of Hematology and Blood Diseases Hospital Peking Union Medical College and Chinese Academy of Medical Sciences No. 288 Nanjing Road 300020 Tianjin China

**Keywords:** ALL, BCR‐ABL, DAPK1, Pin 1, protein–protein interaction

## Abstract

Philadelphia chromosome (Ph)/BCR‐ABL‐positive (ph^+^) ALL is the most common genetic abnormality associated with ALL and has been shown to confer the worst prognosis to both children and adults. Increasing evidence has revealed that the dysregulation of prolyl isomerase Pin 1 contributes to multicancer development and progression, including ALL, although the underlying molecular mechanisms remain unclear. Here, we report that the expression of Pin 1 was enhanced in ph^+^ ALL patient samples and was associated positively with the expression of BCR‐ABL. Genetically or pharmacologically inhibiting Pin 1 expression or activity produces potent therapeutic efficacy against ph^+^ ALL. We further demonstrated that BCR‐ABL enhances the prolyl isomerase activity of Pin 1 by decreasing the phosphorylated level of Pin 1 at Ser 71 and interacting with DAPK1. The inhibition of BCR‐ABL activity by imatinib in human ph^+^ ALL cells reduces the prolyl isomerase activity of Pin 1, further suggesting a key role of the newly identified BCR‐ABL‐Pin 1 axis in ph^+^ ALL progression. Thus, the combined suppression of Pin 1 and BCR‐ABL proteins may be exploited as an additional target therapy for ph^+^ ALL.

## Introduction

Acute lymphoblastic leukemia (ALL) is characterized by aberrations in the proliferation and differentiation of lymphoblasts, leading to failure of the normal immune response and decreased production of normal hematopoiesis. Philadelphia chromosome (Ph)/BCR‐ABL‐positive ALL is the most common genetic abnormality associated with ALL and has been shown to confer the worst prognosis to both children and adults 1, 2. Hematopoietic stem cell transplantation (SCT) has been the gold‐standard therapy for the maintenance of complete remission (CR) in Ph^+^ ALL patients. The prognosis of Ph^+^ ALL patients has dramatically improved upon the approval of BCR‐ABL tyrosine kinase inhibitor (TKI) imatinib mesylate as first‐line treatment. Despite these advances, the prognosis for Ph^+^ ALL patients remains very poor in both children and adults because relapse frequently occurs after SCT or TKI treatment. It was reported that all‐trans retinoic acid (ATRA), medication used for the treatment of acute promyelocytic leukemia, induced cellular differentiation and CD38 expression to inhibit the acquisition of BCR‐ABL mutations for leukemia‐acquired resistance 3, suggesting the potential benefit of ATRA or targets of ATRA in treating Ph^+^ ALL.

Proline‐directed protein phosphorylation is a common and central signaling mechanism that plays crucial roles in diverse cellular processes and controls cell proliferation and transformation, and its dysregulation contributes to many human cancers 4. The peptidyl‐prolyl Pin 1 cis/transisomerase was discovered as an enzyme that specifically recognizes and binds to phosphorylated serines or threonines preceding a proline (phosphor Ser/Thr‐Pro) residue, inducing conformational changes of phosphoproteins 5. Pin 1 also transduces phosphorylation signaling by affecting its substrates’ functions, including protein stability, catalytic activity, phosphorylation status, protein–protein interactions, and/or subcellular localization 6, 7. Pin 1 alterations have been implicated in the amplification of oncogenic signals by stabilizing oncoproteins and/or destabilizing or inactivating tumor suppressors 8, as also shown by its frequent deregulation in several human malignancies. Moreover, recent studies have suggested a pivotal role of Pin 1 in T‐ALL and multicancer progression 9. However, how Pin1 participates in the pathogenesis of cancer or is regulated by other oncogenic signaling pathways in leukemia is not known. In this study, we evaluated the possible cross talk between Pin 1 and BCR‐ABL proteins in the ph^+^ ALL context by analyzing ph^+^ ALL patient samples and cell models of Pin 1 or BCR‐ABL stable knockdown. Here, we show that the expression of Pin 1 was enhanced in the ph^+^ ALL patient samples and is positively associated with the expression of BCR‐ABL. Genetically inhibiting Pin 1 expression produces potent therapeutic efficacy against ph^+^ ALL.

Death‐associated protein kinase 1 (DAPK1), a death domain‐containing calcium/calmodulin‐regulated serine/threonine kinase, functions as a positive mediator of apoptosis. Lee et al. 10 reported that DAPK1 phosphorylates Ser71 in the catalytic active site of Pin 1, thereby inhibiting its cellular function. We demonstrated that BCR‐ABL depletion enhances the phosphorylated Ser71 of Pin 1, prolyl isomerase activity of Pin 1, and cellular function via interacting with DAPK1 protein. In addition, active Pin 1 is a key target of ATRA in acute promyelocytic leukemia and breast cancer 11, suggesting that ATRA might produce antileukaemic effects in Pin 1‐enhanced ph^+^ ALL. Together, our findings suggest a key role of the newly identified BCR‐ABL‐Pin 1 axis in ph^+^ ALL progression. Thus, the combined suppression of Pin 1 and BCR‐ABL proteins may be exploited as an additional target therapy for ph^+^ ALL.

## Materials and Methods

### Patient samples

Primary cells were freshly obtained from the BM of ALL patients at the Institute of Hematology and Blood Diseases Hospital of PUMC. The clinical features of the patients are listed in Table 1. Informed consent was obtained from all the participants in accordance with the Declaration of Helsinki. All the protocols using human specimens were approved by the Institutional Review Board of Institute of Hematology and Blood Diseases Hospital of PUMC.

**Table 1:** Clinical information of leukemia samples

Name	Age (years)	Sex	Diagnosis	Gene	MRD	Status
1	50	M	PH‐ALL‐ALLO‐PBSCT	Negative	Negative	CR
2	19	M	PH‐ALL	Negative	Negative	NR
3	39	M	PH‐ALL‐ALLO‐PBSCT	Negative	Negative	CR
4	36	M	PH‐ALL	Negative	0.17%	CR
5	26	M	PH‐ALL	Negative	0.07%	PR
6	37	F	PH‐ALL	Negative	Negative	CR
7	34	M	PH+ALL	P190 0.36%	Negative	CR
8	26	M	PH+ALL‐ALLO‐PBSCT	P190 0	Negative	CR
9	34	M	PH+ALL‐AUTO‐PBSCT	P190 0	Negative	CR
10	51	F	PH+ALL	P190 0	Negative	CR
11	16	F	PH+ALL	P210 0	Negative	CR
12	36	M	PH+ALL‐ALLO‐PBSCT‐R‐NR	P210 6.89%	1.55%	NR
13	43	F	PH+ALL	P190 0	Negative	CR
14	35	M	PH+ALL‐SYN‐PBSCT‐R	P190 56.09%	10.10%	R
15	51	F	PH+ALL	P210 0	Negative	CR
16	15	M	PH+ALL‐ALLO‐PBSCT	P210 0	Negative	CR
17	15	M	PH+ALL‐ALLO‐PBSCT	P210 0	Negative	CR
18	47	M	PH+ALL‐AUTO‐PBSCT	P190 0	Negative	CR
19	40	F	PH+ALL‐AUTO‐BM+PBSCT	P190 0	Negative	CR
20	27	M	PH+ALL‐ALLO‐PBSCT	P210 0	Negative	CR
21	18	M	PH+ALL	P190 66.08%	98%	Newly diagnosed
22	18	M	PH+ALL	P190 32.08%	52%	NR
23	34	M	PH+ALL	P190 0.23%	0%	CR
24	51	F	PH+ALL	P210 0	0	CR
25	36	M	PH+ALL	P190 67.47%	88.80%	Newly diagnosed
26	51	F	PH+ALL	P210 0	Negative	CR
27	51	F	PH+ALL	P190 0	Negative	CR
28	50	M	PH+ALL‐ALLO‐PBSCT	P190 0	Negative	CR
29	18	M	PH+ALL	P190 0.42%	Negative	NR

CR, complete remission; PR, partial remission; NR, no response; MRD, minimal residual disease.

### Plasmid construction and recombinant proteins

HA‐tagged DAPK1 was constructed in the pcDNA3.1‐HA vector by standard subcloning. N‐terminal GST‐tagged recombinant DAPK1 and pure GST were purchased from Merck Millipore. Full‐length Pin 1 was subcloned in frame to the pGEX4T‐1 to make a GST fusion protein. Myc‐ or His‐tagged BCR‐ABL was constructed in the pcDNA3.1‐Myc His (‐B) vector by standard subcloning. The Pin 1‐shRNA‐targeting sequence has been reported previously 12. The sequence was subcloned into the pSilencerTM3.1‐H1 hygroexpressing vector (Ambion, TX). A Control shRNA oligonucleotide, which does not match any known human coding cDNA, was used as a control.

### Cell lines and imatinib/ATRA treatment

BV173 (human ph^+^ ALL cell lines), NALM‐1 (human ph^+^ ALL cell lines), NALM‐6 (human ph^−^ ALL cell lines), BaF3 (mouse lymphocytes), and HEK 293T cells (human embryonic kidney cell line) were purchased from the Shanghai Institute of Cell Biology, Chinese Academy of Sciences (Shanghai, China), where they were recently authenticated by STR profiling and characterized by mycoplasma detection and cell vitality detection. BV173, NALM‐1, and NALM‐6 cells were cultured in RPMI‐1640 media (Gibco, Carlsbad, CA) containing 10 % heat‐inactivated fetal bovine serum (FBS, Gibco) and 10 U/mL penicillin‐streptomycin (Sigma, St. Louis, MO) in a humidified incubator at 37°C in 5% CO_2_ and 95 % air. BaF3 cells were grown in RPMI 1640 medium with 10% FBS and 10% WEHI‐3B‐conditioned medium (as a source of murine IL‐3) at 37°C and 5% CO2. Imatinib was purchased from Selleck (Beijing, China), and ATRA was purchased from Sigma (Beijing, China). All these reagents were dissolved in dimethyl sulfoxide (DMSO) and were stored at −20°C. The concentration of DMSO as vehicle control was kept under 0.1% throughout all experiments to avoid cytotoxic effects. Both reagents were used at the following concentrations: ATRA, 5 *μ*mol/L 13; imatinib, 1 *μ*mol/L 14.

### Transfection and cell sorting

To generate BV173 cell populations stably expressing Pin 1 shRNA or BCR‐ABL shRNA, the Con‐shRNA, Pin 1‐shRNA 1/2, or BCR‐ABL shRNA 1/2 plasmids 15 were transfected into BV173 cells with a transfection reagent (Lipofectamine^®^ 2000, Thermo Fisher) according to the manufacturer's instructions. After 24 h of transfection, stable transfectants were selected in medium containing 200 *μ*g/mL hygromycin (Calbiochem, San Diego, CA) for 7 days. After two or three passages in the presence of hygromycin, the cultures were used for experiments without cloning. The retroviral vector MSCV‐IRES‐EGFP was used to generate retrovirus expressing Bcr‐Abl p190, which was used to infect the cell line BaF3. Forty‐eight hours after infection, the cells were sorted for EGFP^+^ population using the BD FACS Vantage system.

### Cell growth and cell apoptosis analyses

Growth curve assays were performed in triplicate and were quantified using the Vi‐Cell XR Cell Viability Analyzer (Beckman Coulter, Indianapolis, IN, USA). The cell apoptotic assay of BV173 cells was analyzed 24 h after treatment with either imatinib, ATRA, imatinib and ATRA, or a negative control. The cells were collected and then were washed twice with PBS, fixed in 75 % cold ethanol for 12 h, stained with Annexin V‐FITC/PI (BD Biosciences, San Jose, CA, USA), and evaluated by flow cytometry (BD Biosciences).

### Western blotting

Western blotting was used to examine Pin 1, BCR‐ABL, and other protein levels of patient samples in this study. The polyvinylidene fluoride membrane containing Pin 1, BCR‐ABL, and other proteins was incubated with primary antibodies (1:1000) and then with secondary antibodies, horseradish peroxidase‐conjugated antibody (1:2000). Proteins were revealed using an enhanced chemiluminescence detection method following to the manufacturer's instructions (Pierce). Semiquantification of the positive bands in the images was performed using Quantity One software (Bio‐Rad Laboratories), with GAPDH as the internal control.

### Immunoprecipitation

The cells were washed three times with phosphate‐buffered saline, harvested, and lysed in co‐immunoprecipitation (CO‐IP) buffer as described previously. Total cell lysate (5 mg protein) was subjected to immunoprecipitation with appropriate antibodies, as indicated, overnight at 4°C with gentle agitation, followed by incubation with protein A/G Plus agarose for 24 h at 4°C. The immunocomplex was washed three times and then was mixed with 2× SDS sample buffer and boiled for 5 min. Coprecipitates or whole‐cell extracts were resolved by SDS‐PAGE and blotted onto PVDF membranes (Millipore) for Western blotting assay.

### GST pull‐down assay

GST fusion constructs of Pin 1 or DAPK1 were expressed in *Escherichia coli* and were purified using glutathione–Sepharose 4B beads (Amersham Bioscience). Equal amounts of GST or GST fusion proteins bound to glutathione–Sepharose beads were incubated with lysates from HEK 293T cells transiently transfected with BCR‐ABL‐His. The beads were washed three times, and interacting proteins were detected by Western immunoblotting.

### Pin 1 activity assay

The level of Pin 1 activity was determined using the SensoLyte^®^ Green Pin 1 Activity Assay kit Fluorimetric (AnaSpec, Inc., Fremont, CA), according to the manufacturer's protocols. Briefly, Pin 1 substrate solution was added with DAPK1‐GST, GST, or BCR‐ABL GST and incubated. Then, the fluorescein signal was read using a Multi‐Mode Microplate Reader System (Perkin‐Elmer, Waltham, MA) at excitation and emission wavelengths of 490 and 520 nm, respectively.

### Cell‐cycle analysis

Cells were cultured for 24 h, fixed, and stained with propidium iodide (PI) and RNase A (Sigma‐Aldrich). PI fluorescence intensity was analyzed by a flow cytometer using FL‐2 channel.

## Results

### High Pin 1 expression is positively associated with ph^+^ ALL and BCR‐ABL

**Figure 1:**
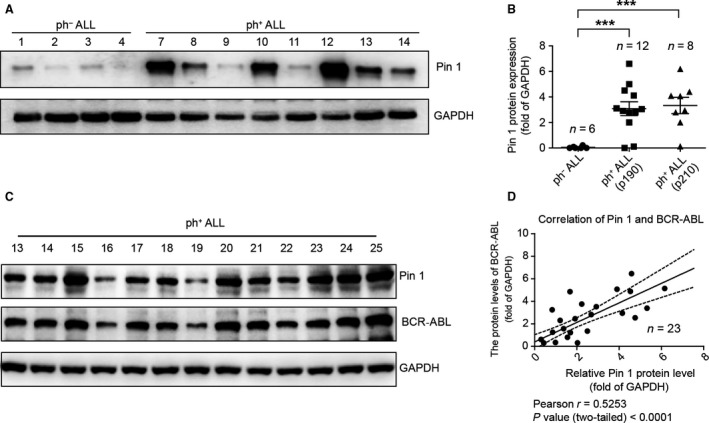
High Pin 1 Expression is Positively Associated with ph^+^ ALL and BCR‐ABL. (A and B) Pin 1 expression was detected by Western blotting (WB) in the BM of ph^+^ ALL and ph^−^ ALL patients (A). The data are the means ± SEM of 3 assays (B). Statistical significance was determined by Student's *t*‐test. ***P < 0.01. (C) The expression of Pin 1 and BCR‐ABL was detected by WB in the BM of ph^+^ ALL patients. The data are representative immunoblots of three assays. (D) Correlation between Pin 1 and BCR‐ABL expressions in ph^+^ ALL patients (*n* = 23). Each point in the plot represents the value from each patient. Statistical significance was measured by Spearman's rank correlation test.

To analyze the putative role of Pin 1 isomerase in the ALL context, we used Western blotting to examine the Pin 1 expression of lymphoblasts from ALL patients (Table 1). Interestingly, we found that the protein level of Pin 1 was much higher in lymphoblast cells from ph^+^ ALL patients than those from ph^−^ ALL patients (Fig. 1A and B). Because the constitutively active tyrosine kinase product BCR‐ABL provided a pathogenetic explanation for the initiation of Ph^+^ ALL as well as a critical molecular therapeutic target, we further detected the expression levels of Pin 1 and BCR‐ABL and explored the potential relationship between these two proteins in ph^+^ ALL patients. The Western blotting data showed that the protein levels of Pin 1 and BCR‐ABL presented a similar tendency in lymphoblasts from ph^+^ ALL patients (Fig. 1C). We further analyzed the correlation of Pin 1 and BCR‐ABL in the ph^+^ ALL context. The statistical results revealed that the protein level of Pin 1 was positively correlated with that of BCR‐ABL in lymphoblasts from 23 ph^+^ ALL patients (Fig. 1D). Taken together, these data indicate that the expression of Pin 1 was enhanced in the ph^+^ ALL patient samples compared with that in ph^−^ ALL patient samples and is associated positively with the expression of BCR‐ABL in these patient samples. The analysis allows us to hypothesize a possible direct relationship between Pin 1 and BCR‐ABL at the protein level in the ph^+^ ALL context.

### Pin 1 depletion induces proliferation inhibition and apoptosis in ph^+^ ALL cells

**Figure 2:**
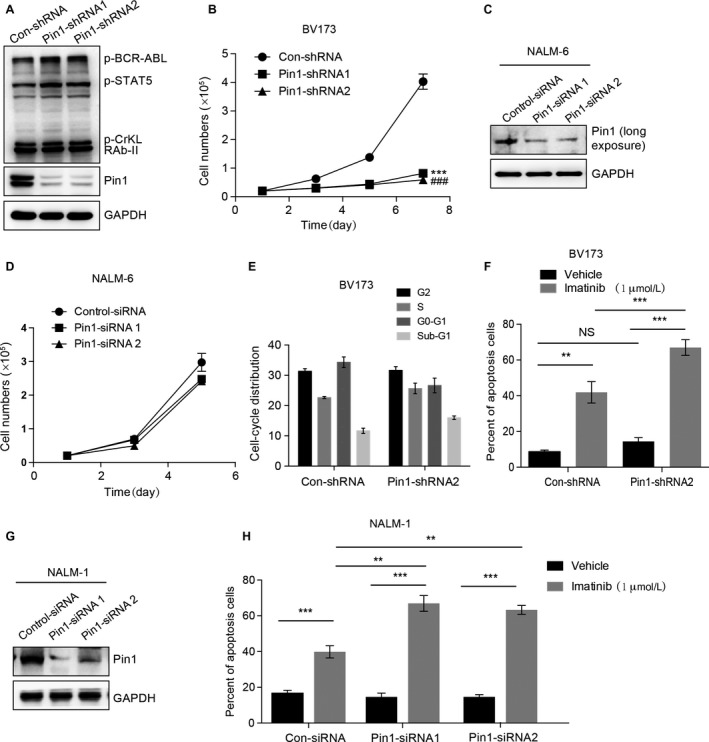
Silencing Pin 1 Induces Proliferation Inhibition in ph^+^ ALL Cells. (A) Silencing Pin 1 does not affect the activity of the BCR‐ABL pathway. The expression levels of p‐BCR‐ABL, p‐STAT5, p‐CrKL, and RAb‐II were detected by WB in BV173 Con‐shRNA and Pin 1‐shRNA1/2 cells. The data are representative immunoblots of three assays. (B) Growth curves of BV173 Con‐shRNA and Pin 1‐shRNA1/2 cells. At different times, BV173 Control shRNA and Pin 1‐shRNA1/2 cells were counted using a Coulter counter. (C and D) Growth curves of NALM‐6 Con‐siRNA and Pin 1‐siRNA1/2 cells. NALM‐6 cell was transfected with Control siRNA or Pin 1‐siRNA1/2 (C). After 24 hours of transfection, NALM‐6 Control siRNA and Pin1‐siRNA1/2 cells were counted using a Coulter counter at different times (D). (E) The effects of Pin 1 depletion to the cell cycle of BV173 cells. BV173 Con‐shRNA and Pin 1‐shRNA2 cells were cultured for 24 h and followed by FACS analysis. (F) Pin 1 depletion increases the sensitivity of the proapoptotic effect induced by imatinib in BV173 cells. BV173 Con‐shRNA and Pin 1‐shRNA2 cells were cultured for 24 h with or without 1 *μ*mol/L imatinib. Cells were stained with Annexin V‐FITC as well as PI and were analyzed by flow cytometry. The percentages of cells staining positive for Annexin V are plotted. (G and H) Silencing of Pin 1 increases the sensitivity of the proapoptotic effect induced by imatinib in NALM‐1 cells. NALM‐1 cell was transfected with Control siRNA or Pin 1‐siRNA1/2 (G). After 24 h of transfection, these cells were cultured for 24 h with or without 1 *μ*mol/L imatinib. Cells were stained with Annexin V‐FITC as well as PI and were analyzed by flow cytometry. The percentages of cells staining positive for Annexin V are plotted (H). For all panels, the data are presented as the means ± SEM of three assays. Statistical significance among groups was determined by one‐way ANOVA; ***P or ^###^ *P* < 0.001, **P < 0.01.

To further investigate the role of Pin 1 in ph^+^ ALL cells, we focused our in vitro study on ph^+^ ALL leukemic cells (BV173). Because BCR‐ABL (p190) is generally accepted to be the factor driving the pathogenesis of ph^+^ ALL and Pin 1 protein is positively correlated with BCR‐ABL in lymphoblasts, we first evaluated whether the absence of Pin 1 could affect the activity of BCR‐ABL in ph^+^ ALL cells. Using the pathscan BCR‐ABL activity assay, we found that the absence of Pin 1 does not affect the activity of the BCR‐ABL pathway, as illustrated by the phosphorylation of BCR‐ABL, Stat5, and CrkL proteins in Pin 1 stable knockdown BV173 cells (Fig. 2A). It was demonstrated that inhibiting Pin 1 expression suppresses cell proliferation and invasion and promotes the apoptosis of carcinoma cells 9, 16. Next, we focused on cell proliferation and apoptosis in BV173 Con‐shRNA and Pin 1‐shRNA 1/2 cells. Using the CCK‐8 cell‐counting kit, we observed a significant decrease in the proliferation of BV173 Pin1‐shRNA 1/2 cells compared with that in Con‐shRNA cells (Fig. 2B). In addition, we detected the proliferation of NALM‐6 cells (Ph^−^ ALL) with or without Pin 1 depletion. The basal‐level expression of Pin 1 is very low in NALM‐6 cells (Fig. 2C), and depletion of Pin 1 didn't affect the proliferation of NALM‐6 cells (Fig. 2D). Furthermore, silencing of Pin 1 didn't affect the cell cycle and induce obvious apoptosis by itself in BV173 cells, which was shown in the Figure 2E and F. However, when Pin 1 silencing was combined with the BCR‐ABL inhibitor imatinib, we observed a significantly higher increase in the apoptosis levels with respect to Con‐shRNA BV173 or Con‐siRNA NALM‐1 cells (Fig. 2F–H), revealing that Pin 1 depletion increased the sensitivity of imatinib treatment in ph^+^ ALL. These data illustrated that Pin 1 depletion might inhibit proliferation and promote imatinib‐induced apoptosis in ph^+^ ALL cells.

### The interaction of BCR‐ABL and DAPK1 enhances the prolyl isomerase activity of Pin 1

**Figure 3:**
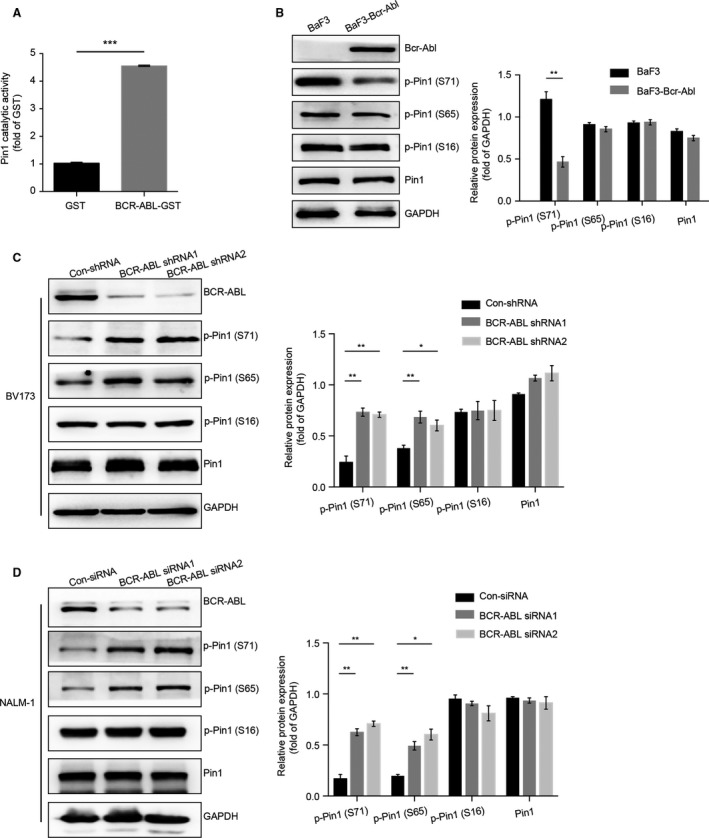
BCR‐ABL Enhances the Prolyl Isomerase Activity of Pin 1. (A) Enhancement of Pin 1 catalytic activity by BCR‐ABL as measured by the PPIase assay. The data are presented as the means ± SEM of three assays. (B) Overexpression of BCR‐ABL inhibited the phosphorylated level of Pin 1. The expression levels of BCR‐ABL, phosphorylated Pin 1 (p‐Pin1‐S71), phosphorylated Pin 1 (p‐Pin1‐S65), phosphorylated Pin 1 (p‐Pin1‐S65), Pin 1, and GAPDH were detected by Western blotting in Bcr‐Abl‐BaF3 versus BaF3 cells. The data are representative immunoblots of three assays. (C and D) The effects of silencing BCR‐ABL to the phosphorylated level of Pin 1 in BV173 and NALM‐1 cells. Cell extracts from the indicated cells were incubated with anti‐BCR‐ABL, phosphorylated Pin 1 (p‐Pin1‐S71), phosphorylated Pin 1 (p‐Pin1‐S65), phosphorylated Pin 1 (p‐Pin1‐S65), Pin 1, and GAPDH Abs. The data represent the means ± SEM of three assays. Statistical significance among the groups was determined by one‐way ANOVA; *P < 0.05, **P < 0.01, ***P < 0.001.

Because the prolyl isomerase Pin 1 regulates ALL progression 9 and there is a positive correlation between Pin 1 and BCR‐ABL in ph^+^ ALL patients, we next investigated whether BCR‐ABL influenced the catalytic activity of Pin 1. Using the in vitro assay of Pin 1 catalytic activity, we found that pure protein of GST‐tagged BCR‐ABL increased Pin 1 catalytic activity compared with the control protein GST (Fig. 3A). Pin 1 catalytic activity can be regulated by the phosphorylation of serine (Ser) 16 and Ser 71, which disrupts or enhances its interactions with its target proteins and is stabilized by the phosphorylation of Ser 65 5, 11. We next examined the phosphorylated levels of Pin 1 in BCR‐ABL‐overexpressed and BCR‐ABL‐silenced cells. The Western blotting data showed that BCR‐ABL overexpression decreased but the absence of BCR‐ABL increased the phosphorylation of Ser 71 of Pin 1, while neither BCR‐ABL depletion nor overexpression affected the phosphorylated level of Pin 1 at Ser 16 (Fig. 3B–D). Interestingly, the absence of BCR‐ABL increased the phosphorylation of Ser 65 of Pin 1 both in BV173 and NALM‐1 cells, but BCR‐ABL overexpression showed no effects on the level of phosphorylated Ser 65 of Pin 1 (Fig. 3B–D). Obviously, it is worth to further dissect the molecular mechanism accounting for the regulation of Pin 1 phosphorylation by BCR‐ABL.

**Figure 4:**
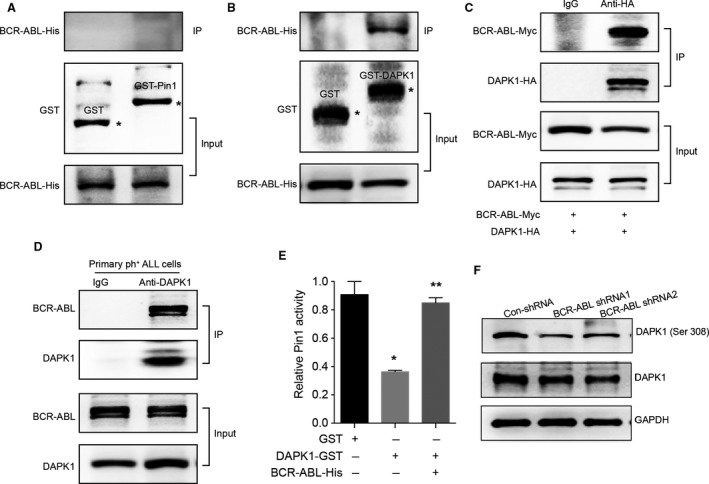
The BCR‐ABL/DAPK1 interaction interferes with the inhibition of Pin 1 catalytic activity by DAPK1. (A) In vitro GST pull‐down assay to verify the interaction of BCR‐ABL (His tag) and Pin 1 (GST tag). Retrieved proteins were evaluated by immunoblotting. GST‐only protein was used as a negative control. GST fusion proteins were stained with Coomassie Blue. The data are representative immunoblots of three assays. (B) In vitro GST pull‐down assay to verify the interaction of BCR‐ABL (His tag) and DAPK1 (GST tag). The retrieved proteins were evaluated by immunoblotting. GST‐only protein was used as a negative control. The GST fusion proteins were stained with Coomassie Blue. The data are representative immunoblots of three assays. (C) The interaction of DAPK1 and BCR‐ABL was evaluated by Co‐IP assay. HEK 293T cells were cotransfected with the indicated constructs of DAPK1‐HA and BCR‐ABL‐Myc. Cell extracts were subjected to IP with IgG or anti‐HA Ab. The data are representative immunoblots of three assays. (D) In vivo CO‐IP assay to verify the endogenous interaction of BCR‐ABL and DAPK1 in primary human ph^+^ ALL cells. Co‐IP of endogenous BCR‐ABL and DAPK1 proteins from fresh primary human ph^+^ ALL cells. Cell extracts were subjected to IP with anti‐DAPK1 Ab or rabbit IgG and blotted with anti‐BCR‐ABL Ab. The data are representative immunoblots of three assays. (E) Inhibition of Pin 1 catalytic activity by DAPK1 was reversed by BCR‐ABL as measured by the PPIase assay. The data are means ± SEM of three assays. (F) Silencing BCR‐ABL decreased the phosphorylated level of DAPK1 at Ser308 in BV173 cells. Cell extracts from the indicated Con‐shRNA, BCR‐ABL shRNA1, and BCR‐ABL shRNA2 BV173 cells were incubated with phosphorylated DAPK1 (Ser308), DAPK1, and GAPDH Abs.

The phosphorylation of Pin1 at Ser 71 by the tumor suppressor DAPK1 inhibits Pin1 catalytic activity and oncogenic function by blocking a phosphorylated substrate from entering the active site 10. BCR‐ABL interacting with critical kinases or adaptor proteins in myeloid cells activates different cellular signaling cascades, such as the Ras pathway, MAPK pathway, Jak‐STAT pathway, and PI3K pathway 17. To investigate the detailed molecular mechanism by which BCR‐ABL regulates Pin 1 activity, we detected the interaction of BCR‐ABL/Pin 1 in vitro, but the data showed that BCR‐ABL displayed no interaction with Pin 1 (Fig. 4A). Unexpectedly, we found that BCR‐ABL directly interacted with DAPK1 using a recombinant GST‐DAPK1 in vitro (Fig. 4B). Moreover, using HA‐tagged DAPK1 (DAPK1‐HA) and BCR‐ABL‐Myc plasmids in cotransfection experiments, we observed that both proteins were reciprocally co‐immunoprecipitated (Fig. 4C). Most importantly, endogenous DAPK1 could also bind BCR‐ABL in the bone marrow freshly obtained from newly diagnosed ALL patients (Fig. 4D). Moreover, Pin 1 activity was examined using an in vitro PPIase assay with or without DAPK1. DAPK1 obviously inhibited Pin 1 activity, and BCR‐ABL protein could reverse the decreased catalytic activity of Pin 1 mediated by DAPK1 (Fig. 4E). We also detected the phosphorylated levels of DAPK1 at Ser308 in the BCR‐ABL‐silenced cells. BCR‐ABL depletion induced dephosphorylation of DAPK1 (Ser308) to activate DAPK1 (Fig. 4F), which may lead subsequent phosphorylation of Pin 1 (Ser71) in Ph^+^ ALL cells. These data indicated that the interaction of BCR‐ABL and DAPK1 enhances the prolyl isomerase activity of Pin 1.

### Combined inhibition of Pin 1 and BCR‐ABL results in antileukemic effects in ph^+^ ALL

**Figure 5:**
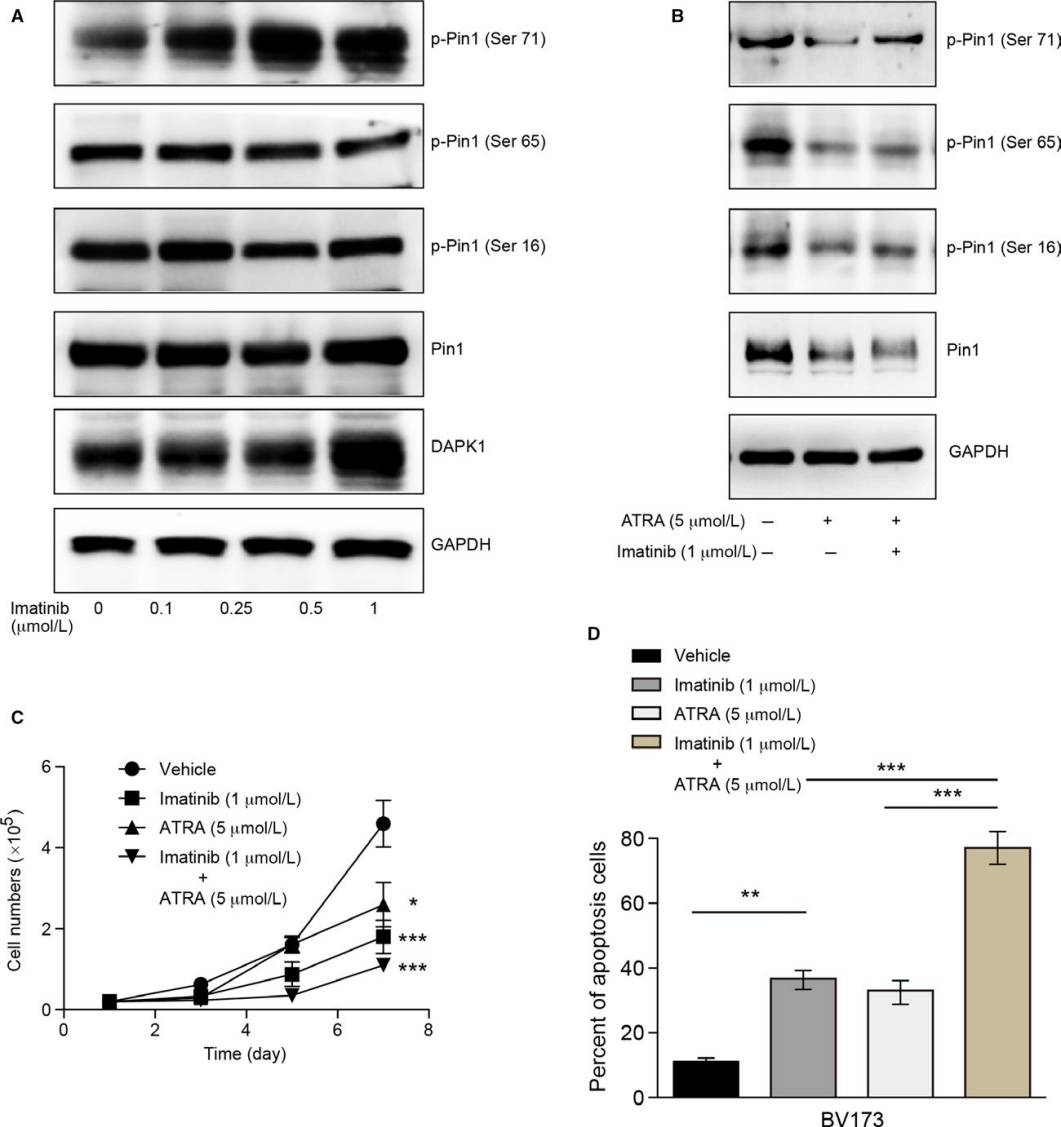
Synergistic effect of imatinib and ATRA on BV173. (A) BV173 cells were treated with the indicated concentrations of imatinib for 24 h. The indicated proteins were detected by immunoblotting. The data are representative blots of three assays. (B) BV173 cells were treated with the indicated concentrations of ATRA and/or imatinib for 24 h. The indicated proteins were detected by immunoblotting. The data are representative blots of three assays. (C) Growth curves of BV173 cells with the indicated treatment. At different times, these cells were counted using a Coulter counter. (D) BV173 cells were cultured for 24 h with the indicated treatment. The cells were stained with Annexin V‐FITC as well as with PI and were analyzed by flow cytometry. The percentages of cells staining positive for Annexin V are plotted. For all panels, the data are representative of the means ± SEM of three assays. Statistical significance among the groups was determined by one‐way ANOVA; *P < 0.05, **P < 0.01, ***P < 0.001.

Consistent with these data, the BCR‐ABL inhibitor imatinib increased the phosphorylation of Pin 1 at Ser 71 in BV173 cells (Fig. 5A), further verifying the effects of BCR‐ABL on Pin 1 activity. All‐trans retinoic acid (ATRA)‐*α* therapy for acute promyelocytic leukemia (APL) is considered to directly and selectively bind, inhibit, and ultimately degrade active Pin 1, thereby exerting potent anticancer activity against APL and triple‐negative breast cancer 11, 18. Accordingly, we observed that ATRA treatment induced the decreased expressions of Pin 1 and phosphorylated Pin 1 at Ser 71, 65, and 16, and combination with imatinib could reverse the decreased phosphorylation of Pin 1 at Ser 71 (Fig. 5B). Moreover, the combination of ATRA and imatinib significantly inhibited the proliferation (Fig. 5C) and enhanced the apoptotic percent of ALL cells (Fig. 5D) compared with the single treatment. Overall, these data suggest that Pin 1 could play a proleukemia role in ph^+^ ALL context, and BCR‐ABL sustained the catalytic activity of Pin 1 by interacting with DAPK1. The combination of Pin 1 and BCR‐ABL inhibitors resulted in further antileukemia effects, which might provide an additional option for ph^+^ ALL therapy.

## Discussion

ALL patients have a higher percentage of induction failure, rate of relapse, and invasion into the central nervous system than most other leukemia patients 19. An important role of Pin 1 signaling in the progression and aggressiveness of solid tumors has been demonstrated 16; in addition, there is increasing evidence supporting the same important role in leukemia 9, 20, 21. In this study, we further demonstrated that the expression of Pin 1 was enhanced in ph^+^ ALL patient samples and is associated positively with the expression of BCR‐ABL. Both genetically inhibiting Pin 1 expression or pharmacologically inhibiting Pin 1 activity by ATRA produces potent therapeutic efficacy against ph^+^ ALL, indicating that Pin1 may act as a new potential target for ph^+^ ALL therapy.

Several mechanisms regulating Pin 1 expression or activity have been recently reported 22, 23. DAPK1 acts as a tumor suppressor to play a rate‐limiting effector in an endoplasmic reticulum (ER) stress‐dependent apoptotic pathway 23. Its expression is epigenetically suppressed in several tumors 24. Lee et al. 10 reported that DAPK1 acts as a kinase responsible for the phosphorylation of Pin 1 on Ser71 in the catalytic active site. Such phosphorylation fully inactivates Pin 1 catalytic activity and inhibits its cellular function. It was also reported that the function of DAPK1 was repressed by Flt3ITD in Flt3ITD^+^ acute myeloid leukemia (AML) 25. In this study, we report, for the first time, that BCR‐ABL influences the catalytic activity of Pin 1 via its interaction with DAPK1. Mechanistically, we show that BCR‐ABL affected phosphorylated Pin 1 at Ser 71. The BCR‐ABL kinase inhibitor imatinib might inhibit the catalytic activity of Pin 1 by increasing the phosphorylation of Pin 1 on Ser 71. Moreover, we observed that BCR‐ABL was responsible for sustained Pin 1 catalytic activity in ph^+^ ALL. Notably, our data not only extended the role of BCR‐ABL in regulating Pin 1 activity but underlined the context of the Pin 1 mechanistic activity.

Functionally, we show that the establishment of the Pin 1/BCR‐ABL relationship may contribute to promote ph^+^ ALL progression. Indeed, more recently, it has been demonstrated that Pin 1 downmodulation by ATRA (all‐trans retinoic acid), known to be used in acute promyelocytic leukemia (APL) therapy, is correlated with tumor cell growth inhibition in APL animal models and human APL cells in vitro as well as in APL patients 11. Notably, this occurs also in triple‐negative breast cancer, thus exerting potent anticancer activity, probably by blocking Pin 1 substrate oncogenes and tumor suppressors at the same time. In keeping with these findings, our work underlines for the first time that Pin 1 isomerase could represent a new potential therapeutic target also in ph^+^ ALL treatment. Overall, our findings may provide a rationale for novel therapy approaches, because the combined inhibition of Pin 1 and BCR‐ABL could suppress aggressive phenotypes, representing a useful tool to interfere with the mechanisms governing ph^+^ ALL cell progression.

## Conflict of Interest

The authors declare that they have no conflict of interest.
